# Intraguild Predation on the Whitefly Parasitoid *Eretmocerus eremicus* by the Generalist Predator *Geocoris punctipes*: A Behavioral Approach

**DOI:** 10.1371/journal.pone.0080679

**Published:** 2013-11-19

**Authors:** María Concepción Velasco-Hernández, Ricardo Ramirez-Romero, Lizette Cicero, Claudia Michel-Rios, Nicolas Desneux

**Affiliations:** 1 Departamento de Producción Agrícola, Centro Universitario de Ciencias Biológicas y Agropecuarias (CUCBA), Universidad de Guadalajara, Zapopan, Jalisco, Mexico; 2 Campo Experimental Mocochá, Instituto Nacional de Investigaciones Forestales, Agrícolas y Pecuarias (INIFAP), Yucatán, Mexico; 3 French National Institute for Agricultural Research (INRA), Sophia-Antipolis, France; University of California, Berkeley, United States of America

## Abstract

Intraguild predation (IGP) takes place when natural enemies that use similar resources attack each other. The impact of IGP on biological control can be significant if the survival of natural enemy species is disrupted. In the present study, we assessed whether *Geocoris punctipes* (Hemiptera: Lygaeidae) engages in IGP on *Eretmocerus eremicus* (Hymenoptera: Aphelinidae) while developing on whitefly nymphs of *Trialeurodes vaporariorum* (Hemiptera: Aleyrodidae). In choice and non-choice tests, we exposed *G*. *punctipes* to parasitized and non-parasitized whitefly nymphs. We found that *G. punctipes* does practice IGP on *E. eremicus*. However, choice tests assessing *G*. *punctipes* consumption revealed a significant preference for non-parasitized *T*. *vaporariorum* nymphs. Subsequently, we investigated whether *E. eremicus* females modify their foraging behavior when exposed to conditions involving IGP risk. To assess this, we analyzed wasp foraging behavior under the following treatments: i) whitefly nymphs only (control = C), ii) whitefly nymphs previously exposed to a predator ( = PEP) and, iii) whitefly nymphs and presence of a predator ( = PP). In non-choice tests we found that *E*. *eremicus* did not significantly modify its number of attacks, attack duration, oviposition duration, or behavior sequences. However, *E*. *eremicus* oviposited significantly more eggs in the PEP treatment. In the PP treatment, *G*. *punctipes* also preyed upon adult *E*. *eremicus* wasps, significantly reducing their number of ovipositions and residence time. When the wasps were studied under choice tests, in which they were exposed simultaneously to all three treatments, the number of attacks and frequency of selection were similar under all treatments. These results indicate that under IGP risk, *E*. *eremicus* maintains several behavioral traits, but can also increase its number of ovipositions in the presence of IG-predator cues. We discuss these findings in the context of population dynamics and biological control.

## Introduction

A ‘guild’ is described as all taxa in a community that use similar resources (food or space) and consequently can compete [1]. Intraguild predation (IGP), in turn, can be understood as attack among natural enemies that use similar resources [2]. IGP can be reciprocal (when both natural enemies attack each other) or asymmetric (when only one species attacks the other). It is generally accepted that IGP is ubiquitous in nature, and several cases have been reported among terrestrial heteropteran predators [3], [4] and aleurophagous predators [5], [6]. For parasitoid insects, IGP is commonly asymmetric, with the parasitoid the hunted natural enemy (IG-prey) and the predator, the ‘true’ predator (IG-predator) that preys on the parasitoid [7], [8], [9], [10], [11], [12]. It has been proposed that IGP can play a role in the persistence or exclusion of participant species [1], [13]. As a result, IGP can be a disruptive or a stabilizing force at the population or community levels [1].

In the biological control context, IGP has been proposed as a factor that can disrupt pest control since the introduction of IG-predators can raise the density of pest herbivores [14], [15]. However, recent analyses have proposed that IGP does not always disrupt biological control [16], [17], and in some cases can even enhance it [18]. Therefore, IGP may or may not influence the success or failure of some biological control programs, and variable effects of IGP on biological control can be expected [16], [19]. For instance Snyder and Ives [8] found that IGP of the beetle *Pterostichus melanarius* Illiger (Coleoptera: Carabidae) on the parasitoid *Aphidius ervi* Haliday (Hymenoptera: Braconidae) disrupts pest control. In contrast, Colfer and Rosenheim [18] found that the control of *Aphis gossypii* Glover (Homoptera: Aphididae) was enhanced when the IG-predator *Hippodamia convergens* Guérin-Méneville (Coleoptera: Coccinellidae) was present jointly with the IG-prey *Lysiphlebus testaceipes* (Cresson) (Hymenoptera: Braconidae).

It is known that some IG-prey are able to avoid IGP [1], and previous studies (e.g. [20]) have shown that IG-prey can avoid areas where predators are present by modifying their behavior. Taylor et al. [21] found that the parasitoid *A. ervi* spent less time foraging on patches previously exposed to the predator *Coccinella septempunctata* L. (Coleoptera: Coccinellidae) and these results were confirmed by Nakashima and Senoo [22] and Nakashima et al. [23]. Modification of behavior by an IG-prey in response to predation risk is though to occur in response to chemical information emitted by an IG-predator and recognized by the IG-prey [24]. The ability to recognize a predator's presence through the perception of direct or indirect signals is an important fitness trait for an IG-prey species [13]. Thus, IG-prey behavior may affect the extent to which IGP influences IG-prey distribution, oviposition behavior, survival [25], [10], and ultimately, biological control effectiveness ([15], [16], [26], but see [18]).

*Trialeurodes vaporariorum* Westwood (Hemiptera: Aleyrodidae) is one of the most harmful insect species for several crops around the world [27], [28], [29], [30]. Females of this species oviposit about 300 eggs over their lifetime and nymphs go through four stages [31], [32]. Whiteflies can cause both direct damage to the plants [33] and indirect damage since they are effective vectors of several plant diseases [34], [35]. *Geocoris punctipes* (Say) (Hemiptera: Lygaeidae), known as the big-eyed bug, is a common natural enemy of whiteflies in the southern United States and Mexico [36], [37]. The big-eyed bug is known to prey on several pests [38], [39], [40], including *Bemisia tabaci* Gennadius and *T*. *vaporariorum* [41], [42], [43]. Adults require a pre-mating period of 2 to 5 days and adult longevity can last up to 112 days [38], [44]. *Geocoris punctipes* is known to exhibit IGP on *Eretmocerus* sp. nr. *emiratus* developing on *B. tabaci* nymphs [9]. *Eretmocerus eremicus* Rose and Zolnerowich (Hymenoptera: Aphelinidae) is a small wasp (∼1 mm) native to the Americas [45]. It is an ecto-endo parasitoid that can parasitize several species of whiteflies, including *B. tabaci*, *T. abutiloneus* Haldeman, and *T*. *vaporariorum* (all Aleyrodidae) [46]. This wasp can parasitize any whitefly nymph instar, but shows preference for second and third instar nymphs [47]. *Eretmocerus eremicus* is commercially available for whitefly control in the Americas and Europe [48], [49], [50].

Both *E. eremicus* and *G. punctipes* are natural enemies of whiteflies and they can be present simultaneously on some crops [51], [52], [53]. Thus, the study of ecological interactions such as IGP between these natural enemies is important to better understand their population dynamics, efficacy and control of related pests [2], [14], [54], [55], [56]. As a first step, to shed light on the interactions among *T*. *vaporariorum*, *E*. *eremicus* and *G*. *punctipes*, we performed several behavioral bioassays under laboratory conditions. The first objective was to assess if *G*. *punctipes* engages in IGP on immature *E*. *eremicus*. A second objective was to determine whether *E*. *eremicus* modifies its foraging behavior when confronted with situations of IGP risk. We hypothesized that female *E*. *eremicus* would reduce foraging behaviors, such as the number of attacks, ovipositions, and residence time, in the presence of IGP risk.

## Materials and Methods

### Plants

Tomato plants were obtained from commercial seeds (var. ‘saladet’) purchased at the Casa del Hortelano (Guadalajara, Jalisco, Mexico). Seeds were sown in plastic pots (9 cm high, 8 cm diameter) containing Nutrigarden® (Sulfatos y Derivados, S.A. de C.V., México) soil and pearlite (Agrolita de México, S.A. de C.V.). Plants were fertilized with “triple 18” fertilizer (SQM Comercial de México S.A. de C.V.) (0.8 g per 1 L water) and grown in a chamber at 24±3°C, 50±10% relative humidity (RH) and a photoperiod of 14∶10 (light: darkness). Plants were used when they reached 5 to 7 leaves of development. Plants were maintained in herbivore-free cages before their use in experiments.

### Trialeurodes vaporariorum

Whiteflies (*T. vaporariorum*) used in the experiments came from a colony maintained at our laboratory and founded with individuals provided by Dr. Carla Sánchez-Hernández (Universidad de Guadalajara, Mexico) and taxonomically verified by the Aleyrodidae specialist Dr. Vicente Carapia (Universidad Autónoma del Estado de Morelos, Mexico). These were virus-free whiteflies.

#### Non-parasitized nymphs

To obtain non-parasitized whiteflies, a tomato plant was placed in a plastic container (60 cm high ×25 cm diameter) with an organdy lid and a sleeve on the side of the cage to introduce insects. Approximately 150 adult whiteflies were introduced in this cage and allowed to oviposit for 48 h. Then the whiteflies were removed, and after a 14-day period, second- and third-instar nymphs were obtained and used in the experiments. We used non-parasitized second- and third-instar nymphs in all of the experiments because *E*. *eremicus* is known to prefer to parasitize these instars [47], [63].

#### Parasitized nymphs

To obtain parasitized whitefly nymphs, we first followed the same procedure as described above for non-parasitized nymphs. Then, after obtaining second- and third-instar nymphs, tomato leaves containing these nymphs were isolated in plastic clip-cages (2.8 cm high ×6.0 cm diameter). Seven couples of the parasitoid *E*. *eremicus* (details below) were introduced into each clip-cage. Parasitoids were allowed to oviposit for 48 h and, after this period, they were removed from the clip cage. After 18–22 days, the presence of parasitoids inside the whitefly nymphs was evident by visual inspection (seeing the parasitoid pupae under a stereo-microscope, DV4 Carl Zeiss), and parasitized nymphs were used in the experiment. Finally, we used parasitized fourth-instar nymphs for the assessment of IGP, because fourth-instar nymphs enable clear assessment of parasitism.

### Eretmocerus eremicus

*Eretmocerus eremicus* wasps were purchased as pupae from Koppert México (Querétaro, Mexico). Upon adult emergence, 15 parasitoid pairs were placed in a petri dish (9 cm diameter) containing a tomato leaflet with 60 to 80 second- or third-instar whitefly nymphs to enable wasps to oviposit before the experiments. Parasitoids were provided with whitefly nymphs before the experiment to avoid egg resorption [57]. Adult wasps were also provided *ad libitum* access to a honey-water solution (1 cm^2^ of paper towel soaked in 7∶3 ml honey: water solution) and tap water (humidifying ∼1 cm^3^ of cotton). The leaflets with whitefly nymphs, honey-water solution, and tap water were replaced every other day. Female parasitoids were used when they were 2 to 4 days old because it is known that they can mate and oviposit when they are 1 day old [57].

### Geocoris punctipes

*Geocoris punctipes* predators were purchased as nymphs from Organismos Benéficos para la Agricultura (Jalisco, Mexico). Nymphs were maintained in polystyrene cages (40 cm length ×30 cm width ×31 cm high) and fed *ad libitum* with ∼5 g of artificial diet [58], tap water (10 ml), commercial pollen (5 g, Apiarios Rancaño, D.F., Mexico), and sorghum seeds (10 g, var. UDG-110, UdG, Mexico) to improve the development of individuals [59], [60]. Artificial diet and water were replaced daily, whereas pollen and sorghum seeds were replaced once a week. Adult predator females were used in experiments when they were 1 to 6 weeks old because they require a pre-mating period of 2 to 5 days and they can live for more than 10 weeks [38].

All insects were maintained at 24±3°C, 50±10% RH, with a photoperiod of 14∶10 (L∶ D), until their use in the experiments.

### Assessment of intraguild predation

Intraguild bioassays were carried out inside a room maintained at 24±3°C, 50±10% RH, and 1800 lux of light intensity. Observations were conducted between 08h00 and 11h00.

No choice bioassay: Observations were performed according to an adaptation of the process described in detail by Naranjo [9]. Overall, a petri dish (9 cm diameter) (hereafter referred to as an arena) with a 1% (m/V) agar layer of 5 mm thickness was used. The agar was covered with filter paper (ISOLAB medium porosity, 8.5 cm diameter) to provide a surface to enable predator movement. In each arena, one of the following treatments was established: 1) 36 non-parasitized nymphs and, 2) 36 parasitized nymphs. To obtain individual nymphs, we used a metal cork-borer (3.5 mm diameter) to cut off the leaf surface surrounding each nymph. When each nymph was isolated, nymphs were placed randomly and equidistantly on the filter paper in the arena. Then, a predator female (previously starved for 24-h) was introduced into the arena. During the starvation period only tap water on a cotton ball (∼1 cm^3^) was provided to the predator. After the introduction of the predator into the arena, it was allowed to forage for 24 h. Nymphs were then observed under a stereo-microscope (DV4 Carl Zeiss) and the number of consumed nymphs (i.e. empty nymphs) was recorded. Each treatment was replicated 14 times, and for each replicate, we used a recently prepared arena and new individuals (nymphs, predators, and wasps). We followed a randomized block design, with time as the blocking factor. Data analysis for this bioassay was done using a non-parametric Mann-Whitney test (U-test) because normality and homoscedasticity assumptions did not fit, even after data transformation.

#### Choice bioassay

In this bioassay, we followed the same procedure described for the non-choice bioassay, with the difference that we set up the arena containing 18 parasitized and 18 non-parasitized nymphs together. In this treatment, the position of each nymph was randomly assigned. To discriminate between parasitized and non-parasitized nymphs, we marked one of the treatments with a non-visible point (using a non-toxic, Sharpie® Utrafine Point Marker) on the back of the leaflet circles. After 24-h, we recorded the number of consumed nymphs and their status (i.e. parasitized or not). This treatment was replicated 14 times, and for each replicate we used a recently prepared arena and new individuals (whitefly nymphs, predators, and wasps).

Free choice bioassay data were analyzed using a Welch two sample *t*-test [61] (model residuals met normality and homoscedasticity assumptions), comparing the mean number of parasitized and non-parasitized prey consumed by *G. punctipes*. Additionally, a preference index (α) that takes into account the consumption of prey over time was used to assess predation on parasitized and non-parasitized whiteflies (for index formula see [9]). This index provides a value between 0 and 1, with 0 indicating a complete preference for non-parasitized prey and 1 indicating a complete preference for parasitized prey. We used a *t*-test to analyze the null hypothesis of non-preference (α = 0.5).

### Parasitoid foraging behavior during IGP risk

Foraging behavior bioassays were carried out inside a room maintained at 24±3°C, 50±10% RH. Wasp behavior was assessed using a digital camera (EOS Digital Rebel XSi Canon©) adapted to a stereo-microscope (DV4 Carl Zeiss) and the software Etholog (2.2) [62].

#### No choice bioassay

Every day, between 08h30 and 12h30, a female parasitoid was introduced in an arena that contained one of the following treatments: 1) Leaflet with whitefly nymphs (hereafter referred to as C), 2) leaflet with whitefly nymphs and previous exposure to predator (hereafter referred to as PEP) and, 3) leaflet with both nymphs and predator present (hereafter referred to as PP). The C treatment consisted of a tomato leaflet containing 60–80 second- and third-instar whitefly nymphs. This leaflet was introduced in a glass vial (5.8×2×1.6 cm) containing humidified filter paper (4×3 cm) and left in the vial for 24 hours prior to the observation. The PEP treatment consisted of a leaflet prepared as described in the treatment C, but a predator was introduced into the vial. This predator was allowed to forage for 24 h prior to the observation of the parasitoid's behavior, and was removed from the vial 10 minutes before the beginning of the observation. Just before the start of the observation, the leaflet containing predator cues was transferred from the vial into the arena. The PP treatment consisted of the leaflet prepared as described for treatment C. However, 10 minutes before the start of the observation, the leaflet was transferred to the arena and a predator female was added. Then, the predator and the wasp were allowed to forage concurrently. In all treatments, the observation period began when a female wasp was introduced into the arena.

For each female wasp, the number and duration of attacks and ovipositions were recorded. Here, we followed observations described by Ardeh et al. [63] to discriminate between attack and oviposition. Namely, if ovipositor insertion lasted up to 50 seconds, it was classified as an attack; if the period was longer than 50 seconds, it was classified as an oviposition. Residence time (i.e., time that the wasp spent foraging on the leaflet in each treatment) and behavioral foraging sequence were also recorded. These response variables were recorded for each wasp during one hour of observation. We used this period of time on the basis of pilot observations and similar published literature [63], [64]. Each treatment was replicated 20 times (i.e. 60 hours of observation). We followed a randomized block design, with time as the blocking factor. For each replicate, we used a recently prepared arena and new individuals to avoid pseudo-replication.

Data were analyzed using linear mixed-effects models (LMM) with the REML method (Restricted Maximum Likelihood estimation) [65]. Response variables were: 1) number of attacks, 2) number of ovipositions, 3) residence time, and 4) duration of attacks. Fixed effects in all four cases were predator treatments (C, control; PEP, previous exposure to predator; and PP, predator presence). Blocks were considered in the random effects, except in the model of residence time (response variable 3), in which the number of ovipositions was considered a random effect. Response variables 1, 2, and 4 were transformed using √x +0.5. In all analyses, multiple comparisons were performed to examine differences among predator exposure treatments, using the ‘estimable’ function of the gmodels package for R statistical software. For the ‘duration of ovipositions’ response variable, a one-way ANOVA was performed. This response variable was transformed using Box-Cox transformation ((y^<lambda>^ - 1)/<lambda>, using <lambda>  = -1) in order to meet model assumptions of normality and homoscedasticity [66]. A Tukey's multiple comparison test was run to compare means among treatments. Additionally, wasp behavioral sequences for each treatment of non-choice bioassay were analyzed and represented graphically. On the basis of previous observations, a foraging behavior list (Table 1) of *E*. *eremicus* on whiteflies was prepared. These behaviors were used for the analysis and ethograms. For the analysis of behavioral sequences we followed the procedure described in detail by Ramirez-Romero et al. [67]. Overall, behavioral transitions were grouped in a global matrix, which was compared with an expected matrix via a G test [68]. To find significant transitions, standardized residual tests were performed. Results of behavioral sequences were represented graphically using ethograms [67], [69].

**Table 1:** Catalogue of behaviors of *Eretmocerus eremicus* analyzed in this study.

Event	Description
Antennation	The wasp touches nymphs with antennae
Attack	The wasp inserts ovipositor under the nymph
Walk	The wasp moves along the leaflet surface
Groom	Any cleaning of the body, including stroking the antennae ovipositor, or wings with the legs or rubbing the legs together
Rest	The wasp stays motionless
Tarsi	The wasp touches nymphs with tarsi
Drag	The wasps drags its ovipositor on the leaflet surface
Feeding	The wasp approaches its head to the nymph, apparently for haemolymph consumption

#### Choice bioassay

**Figure 1:**
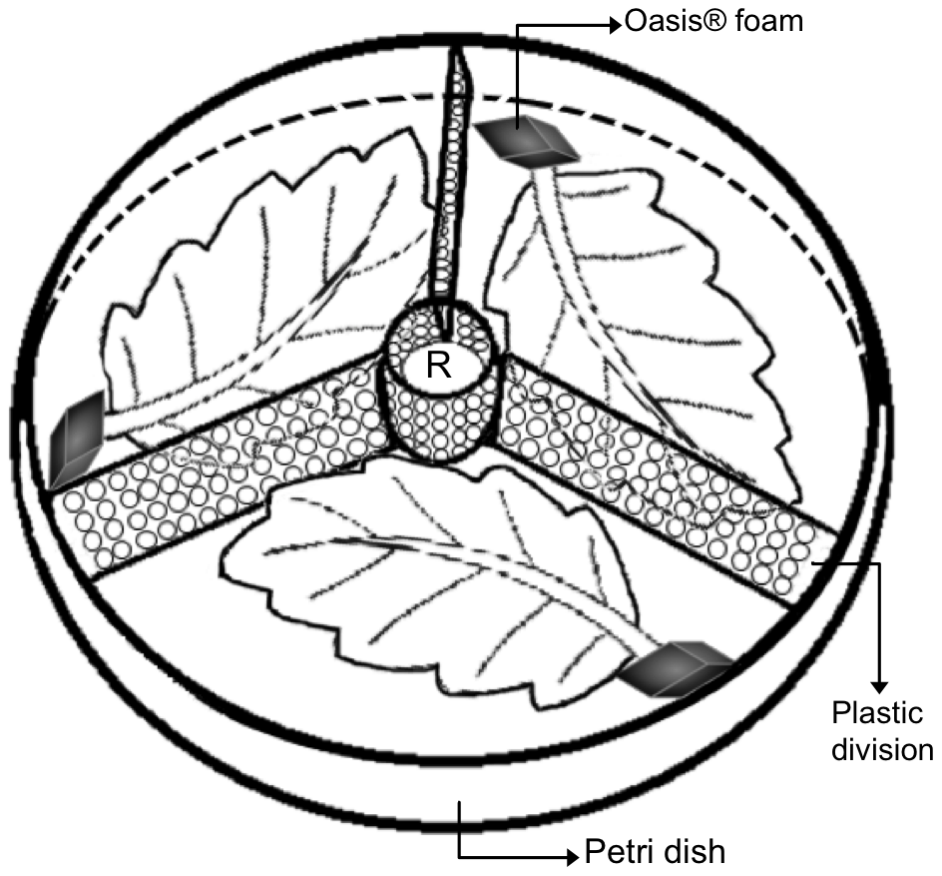
Arena used to study foraging parasitoid behavior during IGP risk (choice bioassay). Wasps were confronted simultaneously with three treatments: i) control, ii) previous exposure to predator and, iii) predator presence. R indicates the wasp release point.

In this bioassay, a female parasitoid was released at the center of a petri dish (hereafter referred to as arena) that contained all three treatments described in the non-choice test (control conditions, previous exposure to a predator, and predator present). This arena had a white bond paper circle (8.5 cm diameter) on the bottom. The three treatments were placed on the paper and were physically separated with three plastic divisions joined at the petri dish perimeter and the center (Figure 1). Each plastic division (3 cm length ×1 cm height) contained 56 holes (2 mm diameter each) that allowed passage of parasitoids but not predators (Figure 1). Leaflets of each treatment were inserted by the stem into wet Oasis® foam pieces (∼1 cm^3^ each) (Nuevo Nova, Smithers-Oasis de Mexico, S.A. de C.V.) to keep leaflets watered. A female parasitoid was introduced in the arena daily between 09h00 and 11h00 and was observed for eight hours as follows. Each female wasp was observed during the first 8 minutes of each sampling hour following a modified variant of the instantaneous sampling method [70]. This procedure was adopted on the basis of pilot observations and other behavioral published works on behavior (e.g. [71]). Each female was observed for a total of 64 minutes and this constituted one replicate. This bioassay was replicated 24 times (c.a. 25 hours of observation). For each replicate, new leaflets and individuals (i.e. whitefly nymphs, predators, and wasps) were used to avoid pseudo-replication. During the 64 minutes, the variables recorded were the number of host attacks and the number of times the wasp selected each treatment.

The number of attacks was analyzed as described in the non-choice bioassay. Proportions of treatment selection were compared using the Marascuilo procedure [72]. Statistical analyses were performed using R, version 2.13.0 [73] and Statistica® 8 software.

## Results

### Assessment of intraguild predation

#### Non-choice bioassay

We found that *G*. *punctipes* readily preys upon parasitized and non-parasitized *T*. *vaporariorum* nymphs. The mean number (±SEM) of consumed nymphs was 32.21 (±1.58) and 33.57 (±0.41) for non-parasitized and parasitized nymphs, respectively. Therefore the mean percentage of consumed nymphs was 89.5% and 93.25% of the non-parasitized and parasitized prey offered, respectively. However, the difference between the two mean numbers of consumed nymphs was not statistically significant (W_(1,27)_ = 122, *P* = 0.271).

#### Choice bioassay

We found that *G. punctipes* significantly prefers to prey on non-parasitized nymphs of *T. vaporariorum* relative to parasitized nymphs when predators were confronted with both nymph forms in a choice arena (*t* = 2.98, df = 24.58, *P* = 0.0064). The mean number (±SEM) of consumed nymphs was 17.78 (±0.28) and 16.42 (±0.35) for non-parasitized and parasitized nymphs, respectively. This result was confirmed by the mean value of the preference index (PI = 0.47), indicating that *G. punctipes* has a significant preference for consuming non-parasitized nymphs (*t* = 2.69, df = 13, *P* = 0.018).

### Parasitoid foraging behavior during IGP risk

#### No-choice Bioassay

**Figure 2:**
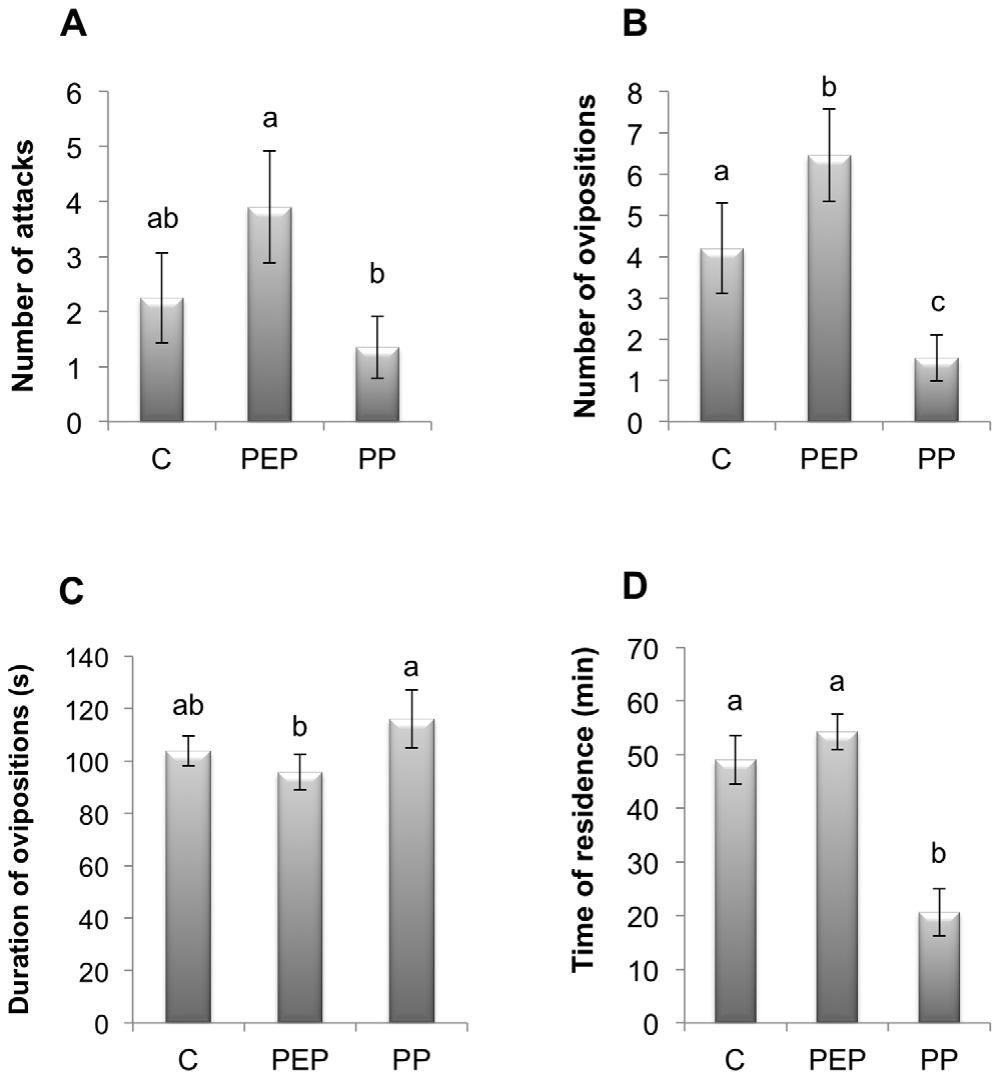
Parasitoid foraging behavior traits of *E*. *eremicus* on *T*. *vaporariorum* nymphs during IGP risk (no choice bioassay). (A) Mean (± SEM) number of attacks. (B) Mean (± SEM) number of ovipositions. (C) Mean (± SEM) duration of ovipositions (in seconds). (D) Mean (± SEM) time of residence displayed by *E*. *eremicus* on leaflets. Observed treatments: C, control; PEP, previous exposure to predator; PP, predator presence. Different letters denote significant differences among treatments (P<0.05).

We found no significant differences in the number of wasp attacks in the C treatment vs. the PEP (*t* = 1.79; df = 38; *P* = 0.08) and PP (*t* = 0.86; df = 38; *P* = 0.39) treatments (Figure 2A). However, significantly more attacks took place in the PEP treatment relative to the PP treatment (*t* = 2.65; df = 38; *P* = 0.011) (Figure 2A). The mean attack duration (in seconds) was not significantly different among treatments (± SEM): C =  27.16 (±2.08), PEP = 26.51 (±1.48), PP =  27.79 (±2.47) (*F*_2,69_ = 0.071, *P* = 0.931).

Significantly more ovipositions took place in the PEP treatment than in the C (*t* = 2.213; df = 38; *P* = 0.032) and PP (*t* = 4.74; df = 38; *P*<0.0001) treatments (Figure 2B). As for oviposition duration, significant differences were found among the treatments (*F*_2,243_ = 4.29, *P* = 0.015) (Figure 2C). Multiple comparisons revealed that oviposition duration was significantly lower in the PEP treatment than in the PP treatment (*P* = 0.032). However, wasp oviposition duration under the C treatment was not significantly different from duration under the PEP (*P* = 0.087) and PP (*P* = 0.594) treatments (Figure 2C). Wasps foraged for significantly less time under the PP treatment relative to the C (*t* = 5.063; df = 44; *P*<0.0001) and the PEP (*t* = 4.826; df = 44; *P* = 0.0001) treatments (Figure 2D).

**Figure 3:**
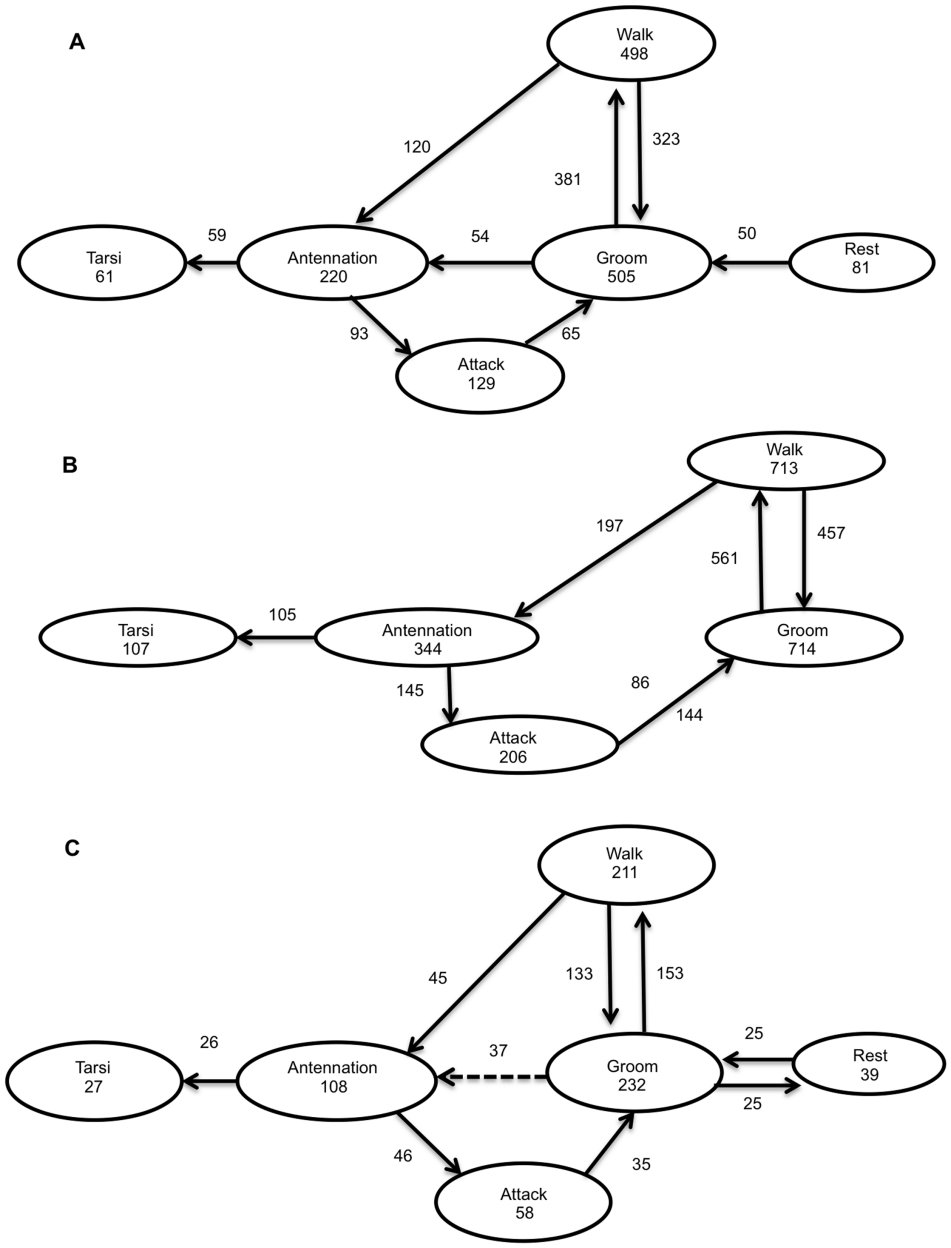
Flow diagrams showing the behavioral sequence of *E*. *eremicus* under IGP risk (no choice bioassay). Three treatments are illustrated: (**A**) control leaflets, (**B**) leaflets with previous exposure to predator and, (**C**) leaflets with presence of predator. Boxes represent behavioral acts, and numbers inside the boxes are behavioral repetitions. Solid arrows represent transitions significantly different from expected (non-random) transitions, and dashed arrows represent transitions not significantly different from expected (random) transitions. Numbers next to arrows represent the numbers of transitions. Transitions with a relative frequency lower than 3% are not shown.

When behavioral sequences were analyzed, it was observed that female parasitoids exhibited a stereotyped behavior (Figure 3). In the ethograms of the C (Figure 3A) and the PP (Figure 3C) treatments, wasps exhibited six main behaviors: *walk*, *antennation*, *groom*, *attack*, *tarsi*, and *rest*. For the PEP treatment (Figure 3B), the *rest* behavior is not shown due to its low relative frequency (<0.03).

Overall, the behavioral sequence under the C treatment (Figure 3A) can be described as follows: the wasp starts *walking* on the leaf and after *walking* the wasp can *groom* (72.9%) or start *antennation* (27.1%). If the wasps *groom*, they proceed to *walk* again (87.6%) or exhibit *antennation* (12.4%). When wasps exhibit *antennation*, they can proceed to *attack* (61.2%) or contact nymphs with *tarsi* (38.8%). After an *attack*, wasps generally *groom* (Figure 3A). Wasps under the PEP treatment (Figure 3B) exhibited the same overall behavioral sequence. However, wasps under the PEP treatment exhibited the *rest* behavior in a low relative frequency (<0.03) and after *groom*, they generally *walked* again (Figure 3B). As for wasps in the PP treatment (Figure 3C), they exhibited a similar behavioral sequence to wasps in the C treatment. However, after *groom*, wasps generally *walk* again (71.2%) or *rest* (11.6%) (Figure 3C).

#### Choice bioassay

There were no significant differences among treatments in the number of wasp attacks (*F*_2,56_ = 0.94, *P* = 0.396). The mean numbers of attacks by *E*. *eremicus* females (± SEM) were: C = 0.875 (±0.30), PEP = 0.958 (±0.414) and PP = 0.875 (±0.296). There were no significant differences between the proportion of selections that *E. eremicus* made for the C treatment (0.58) and the proportion of selections for PEP (0.37) (*X* ^2^ = 0.345, *P*>0.05) or PP (0.75) treatments (*X* ^2^ = 0.328, *P*>0.05). However, the proportions of selections for PP vs. PEP treatments were significantly different (*X* ^2^ = 0.325, *P*<0.05).

Finally, in both choice and non-choice tests we observed that adult wasps were preyed on when the predators were present (PP treatment). However, in the non-choice test, the percentage of preyed-upon wasps (90%) was significantly higher (*X* ^2^ = 7.607; df = 1; *P* = 0.005) than the percentage (45%) in the choice tests.

## Discussion

Both *E. eremicus* and *G. punctipes* are natural enemies of whiteflies that can be present at the same time on some crops [51], [52], [53]. Thus the study of their ecological interactions is important for improving our understanding of their population dynamics, efficacy, and control of related pests [2], [14], [54], [55], [56]. Our results showed that *G*. *punctipes* engages in IGP on *E*. *eremicus* developing on *T*. *vaporariorum* nymphs. In addition, *G*. *punctipes* prefers feeding on non-parasitized nymphs to parasitized nymphs. When we analyzed *E*. *eremicus* foraging behavior under IGP risk, we found that, overall, wasps did not modify most of the analyzed foraging behavior traits. However, wasps in the PEP treatment exhibited more ovipositions relative to the control. As for the PP treatment, we found that under non-choice tests, 90% of the wasps were consumed by the predator, significantly reducing the number of ovipositions and residence time relative to the control. Nevertheless, under choice tests, none of the behavioral traits were significantly different among treatments, and significantly fewer (45%) adult wasps were preyed upon.

### Assessment of intraguild predation

Several cases of IGP have been reported for generalist heteropteran predators [3], [4] and aleurophagous predators [5], [6]. *Geocoris punctipes* is known to be a generalist predator [36] and has been previously reported as an IG-predator on *E*. sp. nr. *emiratus* developing on *B. tabaci* nymphs [9]. Thus, it was expected that this predator could exhibit IGP on the wasp *E*. *eremicus* developing on *T*. *vaporariorum* nymphs. As expected, our results showed that this predator engages in IGP on the parasitoid pupae. We found that under non-choice tests *G. punctipes* prey on parasitized and non-parasitized whitefly nymphs at a similar rate. However, in choice tests the predator exhibited a preference for non-parasitized whitefly nymphs. This preference for non-parasitized prey has been previously reported for other IG-predators [18], [74]. Nevertheless, in a previous study of *G*. *punctipes*, Naranjo [9] showed that this IG-predator preferred parasitized nymphs to non-parasitized nymphs. This author hypothesized that the stronger appearance (in terms of color and size) of parasitized nymphs could be behind the IG-predator preference. Following this postulate, in our experimental set up, the IG-predator should have preferred the parasitized 4^th^ instar nymphs, more obvious (i.e., larger) than non-parasitized 2^nd^–3^rd^ instar nymphs. However, this was not the case, and actually *G*. *punctipes* preferred non-parasitized nymphs. These results indicate that some other factor(s) besides appearance must be influencing IG-predator preferences. Indeed, besides prey appearance, other potential factors influencing predator preference include mechanical aspects (including hardening of the cuticle) [12], [75], physiological/chemical changes [76], [77], and prey species [78], [79], [80]. Further studies aimed at understanding how these factors act on IG-predator preferences are warranted.

### Parasitoid foraging behavior during IGP risk

Another important factor influencing the effects of IGP on prey population dynamics is the behavioral response of the IG-prey in the presence of the IG-predator [81], [82]. Previous studies have reported that IG-prey behavioral traits such as host encounter, attack and patch residence times are reduced under treatments where IGP risk is present [20], [83]. It has been proposed that these behavioral modifications may be related to parasitoid detection of predator cues [20]. Some parasitoids such as *A. ervi* use the IG-predator cues to avoid IGP [23]. In our biological model, *G*. *punctipes* may produce cues during foraging [41], [84]. Therefore, it would be advantageous to *E*. *eremicus* to detect the predator cues and modify its behavior to avoid IGP. For this reason, we initially hypothesized that *E*. *eremicus* would reduce foraging behaviors such as patch time residence and the number of attacks and ovipositions in the presence of IGP risk. However, our results did not support this hypothesis (see Figures 2A, 2C, and 3). In addition, when predator cues were present (PEP treatment), the number of wasp ovipositions was higher relative to the control (Figure 2B). Although unexpected, these results agree with other studies reporting heterogeneous behavioral responses of IG-prey confronted with IGP risk [10], [21], [83]. This indicates that behavioral responses of IG-prey species under IGP risk may not always be modified [20], [23] to avoid IGP risk. A possible explanation is related to the trade-off in behavioral decisions that the wasp can make when facing a high quality patch combined with IGP risk. In our experimental setup, wasps were provided with a relatively high number of good quality hosts (non-parasitized, 2^nd^–3^rd^ instar nymphs, which are preferred for parasitism [47], [63]). It is possible that even with the IGP risk, the wasp decides to continue foraging and ovipositing on these patches. This possibility is in line with the idea of an optimal wasp response in which the profit of staying on the patch surpasses the risk of being preyed upon, so the wasp remains on the patch [85]. This may explain why the wasp exhibit similar behavioral traits under IGP risk compared with control patches. However, it is not clear why the wasp oviposits more in the presence of IG-predator cues. Perhaps, the wasp increases its ovipositions (patch exploitation) in the presence of IG-predator cues to compensate for the IGP risk. Several behavioral modifications can take place under predation risk [86] including those related to reproduction [87]. Further studies are required to understand the factors driving *E*. *eremicus* responses under IGP risk, particularly those leading to increase oviposition rates, a response previously displayed by other IG-prey species [7], [21], [88].

While observing the PP treatment in non-choice tests, we found that the big-eyed bug frequently preyed upon adult *E*. *eremicus* (90% of potential prey attacked). As mentioned before, we found no evidence of attempts by the wasp to avoid the leaflets where the predator was present. In spite of prey-predator encounters, wasps exhibited similar foraging behavior to those in the control group. Nevertheless, the number of ovipositions and residence time under the PP treatment were significantly reduced relative to the control. This was a result of the high and relatively rapid (∼20 minutes) rate of wasp predation in the PP treatment. Thus, our results in non-choice tests suggest that *E*. *eremicus* may continue to forage regardless of the risk of predation.

When *E*. *eremicus* was confronted with predators in a choice test, the number of attacks by the wasps and patch frequency selection were similar in all treatments. This supports the results found in the non-choice test indicating that *E*. *eremicus* does not modify its foraging behavior under IGP risk. However, under choice tests it was observed that adult predation was significantly lower relative to the non-choice test (45% vs. 90%). During observations, it was noticed that wasps moved to predator-free zones when prey-predator encounters occurred. Thus, while our results suggest that in free choice situations, *E*. *eremicus* will not modify its foraging behavior in response to predation risk, if prey-predator encounters do occur, the wasp will tend to move to predator-free zones and thereby reduce the rate of predation.

Both *E. eremicus* and *G. punctipes* are natural enemies of whiteflies that are commercially available in the United States and Mexico ([34], [48], [89], Méndez JM, Org. Benef. Occ. SA de CV, personal communication), and they can be present at the same time on some crops [51], [52], [53]. It is broadly accepted that IGP can influence the population dynamics of implicated species and biological control programs [14], [15], [16], [17], [18], [54], [55]. Therefore, knowledge gained on IGP interactions can be useful for such programs. The ability of *G*. *punctipes* to prey on immature and adult *E*. *eremicus* observed in our study suggests that the presence of both natural enemies may be detrimental for the wasp due to IGP. However, the observed IG-predator preference for non-parasitized *T*. *vaporariorum* nymphs and IG-prey behavior (particularly, the increased rate of oviposition when predator cues are present) could mitigate a potential IGP impact [18]. In view of these results, it is necessary to evaluate the extent to which IGP on *E*. *eremicus* by *G*. *punctipes* affects population dynamics under semi-field or field conditions. It is well-known that, under field conditions, other factors such as complexity of the habitat, prey time of exposition, and escape possibilities can affect IGP outcomes [90], [91]. At present, it would seem sub-optimal to use the two natural enemies together.

## Conclusions

In the current study, we first showed that, as expected, the predator *G*. *punctipes* engages in IGP on the wasp *E*. *eremicus*. To our knowledge this is the first report of IGP on both immature and adult *E*. *eremicus* by *G*. *punctipes*. In addition, the IG-predator exhibited a preference for preying on non-parasitized rather than parasitized nymphs. Furthermore, contrary to our initial hypothesis, the IG-prey *E*. *eremicus* did not reduce its foraging behavior traits under IGP risk. However, under non-choice situations, the wasps did exhibit increased oviposition on leaflets with predator cues. In view of these results, semi-field and field bioassays are warranted to further assess the extent to which IGP and IG-prey behavior can modulate the population dynamics of species considered in our study.
